# Evaluation of Whole Brain Radiotherapy among Lung Cancer Patients with Brain Metastases in Relation to Health Care Level and Survival

**DOI:** 10.3390/life12040525

**Published:** 2022-04-01

**Authors:** Gabriella Frisk, Maria Helde Frankling, Anna Warnqvist, Linda Björkhem-Bergman, Mattias Hedman

**Affiliations:** 1 Department of Neurobiology, Care Sciences and Society (NVS), Division of Clinical Geriatrics, Karolinska Institutet, 141 83 Huddinge, Sweden; maria.helde.frankling@ki.se (M.H.F.); linda.bjorkhem-bergman@ki.se (L.B.-B.); 2 ASIH Stockholm Södra, Palliative Home Care and Specialized Palliative Ward, 125 59 Älvsjö, Sweden; 3 Karolinska University Hospital, Thoracic Oncology Center, Theme Cancer, 171 64 Solna, Sweden; 4 Department of Environmental Medicine, Division of Biostatistics, Karolinska Institutet, 171 77 Stockholm, Sweden; anna.warnqvist@ki.se; 5 Department of Oncology-Pathology, Karolinska Institute, Karolinska University Hospital Solna, 171 76 Stockholm, Sweden; mattias.hedman@regionstockholm.se

**Keywords:** brain metastases, gender, health care level, lung cancer, palliative care, whole-brain radiotherapy

## Abstract

Whole-brain radiotherapy (WBRT) as a treatment for brain metastases has been questioned over the last years. This study aimed to evaluate health care levels and survival after WBRT in a cohort of lung cancer patients with brain metastases receiving WBRT in Stockholm, Sweden, from 2008 to 2019 (*n* = 384). If the patients were able to come home again was estimated using logistic regression and odds ratios (OR) and survival by using Cox regression. The median age in the cohort was 65.6 years, the median survival following WBRT was 2.4 months (interquartile range (IQR) 1.2–6.2 months), and 84 (22%) patients were not able to come home after treatment. Significantly more males could come home again after WBRT compared to women (OR = 0.37, 95%CI 0.20–0.68). Patients with performance status scores WHO 3–4 had a median survival of 1.0 months, hazard ratio (HR) = 4.69 (95%CI 3.31–6.64) versus WHO score 0–1. Patients admitted to a palliative ward before WBRT had a median survival of 0.85 months, HR = 2.26 (95%CI 1.53–3.34) versus being at home. In conclusion, patients treated with WBRT had a short median survival and 20% could not be discharged from the hospital following treatment. Significantly more women did not come home again.

## 1. Introduction

Lung cancer is the most common cancer causing brain metastases at the time of diagnosis [1] and brain metastases can be found in up to 50% of patients with lung cancer [2,3]. Brain metastases among lung cancer patients have, in general, been associated with serious symptoms [4,5] and a short survival [1]. However, local treatments of brain metastases have been developed over time in terms of refined neuroimaging, surgery, and radiotherapy [6]. This allows patients to receive improved treatment with prolonged benefits and fewer side effects [7]. In addition, there have been advances in systemic oncological therapies for lung cancer patients in recent years, and systemic treatment, including chemotherapy, targeted therapy, and immunotherapy, may prevent or delay brain metastases [8,9,10]. Determining the appropriate treatment for patients with lung cancer and brain metastases, therefore, requires a clear understanding of the brain metastases, molecular characteristics, tumor histology, and the overall lung cancer prognosis [11].

Overall survival from the date of diagnosis of brain metastases in lung cancer is short and varies from a few months up to just over one year in former studies [12]. Patients with brain metastases at the time of diagnosis have a median survival of 4–10 months [1]. The most favorable prognostic factors for lung cancer patients with brain metastases seem to be young age (under 50 years), good performance status (Karnofsky performance score between 90 and 100), absence of extracranial metastases, and a low number of brain metastases [7,13]. Moreover, patients with adenocarcinomas with positive epidermal growth factor receptor (EGFR) and anaplastic lymphoma kinase (ALK) alterations show improved survival compared to those without these alterations [13,14]. Several scoring systems have been developed to prognosticate survival in patients with brain metastases and thus aid clinicians in treatment decisions regarding WBRT: Radiation Therapy Oncology Group (RTOG) recursive partitioning analysis (RPA) [15], graded prognostic assessment (GPA) [16], and diagnosis-specific GPA (Lung-molGPA score) that includes assessment of EGFR and ALK alterations in non-small cell lung cancer (NSCLC) [11,17]. These scoring systems provide useful tools for patients with good prognoses while avoiding overtreatment in patients with poor prognoses [18]. 

Patients with few and small size brain metastases due to lung cancer can be treated with surgery, sometimes followed by adjuvant radiotherapy, or with stereotactic radiotherapy [7,16,18]. Until recently, whole-brain radiotherapy (WBRT) was the primary treatment when aiming for symptom control for patients with poor prognosis, poor performance status, massive distribution of brain metastases, and uncontrolled spread of other distant metastases. However, the use of WBRT has decreased in recent years [7] for many reasons, including the development of other more localized treatments for brain metastases and due to a concern about late side effects due to WBRT. There are also better tools to select patients who will benefit from the WBRT and negative results from randomized trials [7,10,19,20]. Thus, it has been argued that patients with the more advanced disease, according to prognostic scores, should not be treated with WBRT [21]. Still, updated consensus guidelines are lacking [22] and treatment practices vary greatly in a European setting [23,24].

According to the Swedish national treatment guidelines, chemotherapy is the first treatment of choice for small cell lung cancer (SCLC) patients with brain metastases at diagnosis. Patients with NSCLC and solitary brain metastases should be discussed for the possibility of radical treatment with neurosurgery; otherwise, stereotactic radiotherapy (SRT) or radiosurgery with a gamma knife. Patients with multiple brain metastases from NSCLC should primarily be treated with steroids and thereafter be discussed for WBRT. Performance status and expected survival should be taken into account in treatment decisions regarding WBRT [25]. 

If WBRT is given to terminal patients, it affects the end-of-life period, including the patient’s ability to choose health care level in this situation. Deaths in hospitals are common among cancer patients in Western countries, even though many of them prefer their homes as the place for dying [26]. 

Palliative care is a specialized medical care for patients with incurable diseases and life-threatening illnesses [27]. Palliative care aims to maintain as high a quality of life (QoL) as possible and to relieve symptoms. The goal is not necessarily to prolong life neither to hasten death. As maintained, QoL is the goal, but the side effects of medical treatments and interventions should not outweigh the possible beneficial effects, and the focus should always remain on symptom relief. This must be considered when making treatment decisions regarding WBRT.

Region Stockholm in Sweden had a population of 2,377,081 in 2019 [28]. The health care system in Sweden is funded with taxes and Karolinska University hospital is a tertiary healthcare center in the region providing for medical oncology, neurosurgery, and radiotherapy. The region has an expanded network of advanced palliative home care units for cancer patients as well as several specialized palliative wards. 

The lung cancer incidence in Sweden has increased by 30% in the last 15 years [25], although Sweden has a very low prevalence of smokers [29]. In 2020, 4325 patients were diagnosed with lung cancer in Sweden [30,31], approximately 1000 of these new cases were from the Stockholm region [32]. In Sweden, the expected relative 5-year survival rate after diagnosis is 20% (17% in men, 24% in women) [33]. In a recent Swedish cohort study on lung cancer patients (*n* = 3562) with access to advanced palliative care, 52% of the patients had at least one hospital admission during the last month of life, and 20% of patients died in hospital [34]. 

This study aimed to evaluate health care level and survival following treatment with WBRT. To this end, a population-based sample of 384 lung cancer patients with metastases to the brain was analyzed, and the level of care before, during, and after treatment was assessed. To our knowledge, this is the first time health care level in relation to treatment with WBRT in patients with metastatic lung cancer has been studied. 

## 2. Materials and Methods

### 2.1. Study Population and Patient Characteristics

In this cohort study, 474 lung cancer patients with brain metastases who were treated with WBRT from 2008 to 2019 at the Karolinska University Hospital in Stockholm, Sweden, were identified. In the analysis, we included patients with all different histological types of lung cancer, with different numbers of brain metastases, and they could be located both in the cerebrum or cerebellum. We also included a small number of patients who had received other local treatments before WBRT. However, 90 patients were excluded as they did not fulfill the inclusion criteria: They had other cancer diagnoses (*n* = 5), metastases to the eyes (*n* = 12), or the scalp (*n* = 11). Patients who received adjuvant radiotherapy following surgery of metastases (*n* = 22), stereotactic treatment in a smaller part of the brain (*n* = 6), or prophylactic treatment (*n* = 31) were excluded from the study. The final cohort included 384 patients. 

Through detailed reviews of the medical files, we gathered information on clinical and biological factors from the lung cancer, including date of primary lung cancer diagnosis, Tumor-Node-Metastases (TNM)-classification at the time of diagnosis, tumor histology, epidermal growth factor receptor (EGFR), anaplastic lymphoma kinase (ALK), and Kirsten rat sarcoma virus (KRAS) -status. We also gathered information on oncological treatment (neoadjuvant/adjuvant), date of first distant metastases (if other than the brain), number and type of oncological palliative treatments, and the date when brain metastases due to lung cancer were diagnosed. The date of start of WBRT, performance status (PS) according to WHO (score 0–5) and health care level (home, hospital, or specialized palliative care ward) the week before treatment with WBRT, the dose of radiotherapy in Gray (Gy) and the patient’s marital status (cohabitation or not and living with children at home or not) were also collected. The PS, according to WHO, was extracted from the electronic medical files. In those cases where PS was not noted in the medical records, it was assessed retrospectively using documented information on the patient’s functional level. The PS WHO score is a scale from 0–5, where zero means that the patient is fully active, three means that the patient is capable of only limited self-care, confined to bed or chair for 50% or more of waking hours, and five means that the patient is dead [35]. From here on, this score is referred to as the “WHO score”.

The radiotherapy treatment was delivered using 6 Mega Volt (MV) photons and two opposed fields. Clinical target volume (CTV) consisted of the brain defined by the skull and limited inferiorly by vertebra C1. 

### 2.2. Outcome Information

The primary outcome in this study was the date of death of any cause after WBRT in this cohort of lung cancer patients with brain metastases. The secondary outcome was to analyze if the patients were able to come home after the treatment with WBRT or not.

### 2.3. Statistical Analyses

All variables were presented as total numbers (percentage). The median survival time was calculated from the start of WBRT using the Kaplan–Meier estimator, and Cox proportional hazards regression models were used to estimate and compare the time to death by the clinical and biological characteristics with hazard ratios (HR) and 95% confidence intervals (CI). In the multivariable analyses, adjustment was made for age at WBRT in intervals and calendar period of WBRT in intervals. In a secondary multivariable model, adjustment was also made for the WHO performance status score. The proportional hazards assumption was formally tested based on Schoenfeld residuals from the Cox model and found to be satisfied, except for the model with EGFR status. This model was stratified by the treatment interval, after which the proportional assumption was fulfilled. A logistic regression model was used to identify risk factors for not coming home again after treatment with WBRT, using odds ratios (OR) and 95% CI. This model was also adjusted for age at WBRT in intervals and for calendar period of WBRT in intervals at first, and in a second model also for WHO performance status score. As an ad hoc analysis, we also looked at the subgroups treated with SRT or surgery in the history before WBRT. The sample sizes were small (38 and 14 patients, respectively) and the analyses were simplified accordingly. The empirical survival curves were visualized for both groups. The counts and percentages were calculated for different variables of interest (as in Table 1) in both subgroups. Minimum, maximum, and median survival times were also counted. For the SRT group, the univariate Cox models were estimated using the different variables of interest. 

The analysis was done in Stata, version 15 (StataCorp. 2017 Stata Statistical Software: Release 15. College Station, TX, USA; StataCorp LLC.). *p*-value < 0.05 was considered as statistically significant. 

## 3. Results

### 3.1. Clinical Characteristics of the Cohort

The median age at the time for treatment with WBRT was 65.6 years (range 36–91 years). At the time of the primary lung cancer diagnosis, 177 patients (46%) had brain metastases, and 207 patients (54%) developed brain metastases later during their disease trajectory. Regarding the histology of the primary lung cancer, 265 (69%) had adenocarcinoma, 63 (16%) SCLC, and 26 (7%) squamous cell carcinoma. The palliative oncological treatment lines before WBRT were in median 1 (range 0–6). Fifty-two patients (14%) had thyrosinkinase inhibitors before WBRT and 331 patients (86%) had no adjuvant therapy. Eight (2%) patients had immunotherapy before WBRT (Table 1). 

### 3.2. Brain Metastases and WBRT

At the time-point for treatment with WBRT, 300 patients (78%) had massive brain metastases, whereas 45 patients (12%) had 1–3 metastases, and 37 (10%) had one single metastasis to the brain. Most of the patients (*n* = 356, 93%), were treated with 4 Grey (Gy) x 5 and 22 (6%) with 3 Gy x 10. All patients were treated with steroids (Table 1). Median survival when treated with 4 Gy x 5 was 2.4 months, and when treated with 3 Gy x 10, it was 3.5 months. There was no statistically significant difference between the groups (Table 2).

### 3.3. Overall Survival

**Figure 1:**
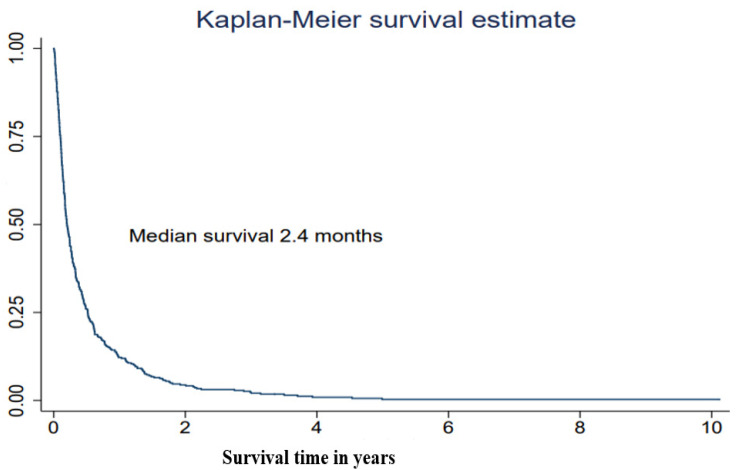
Survival after treatment in a cohort of lung cancer patients with brain metastases receiving whole-brain radiotherapy (WBRT), in Stockholm, Sweden 2008–2019 (*n* = 384).

**Figure 2:**
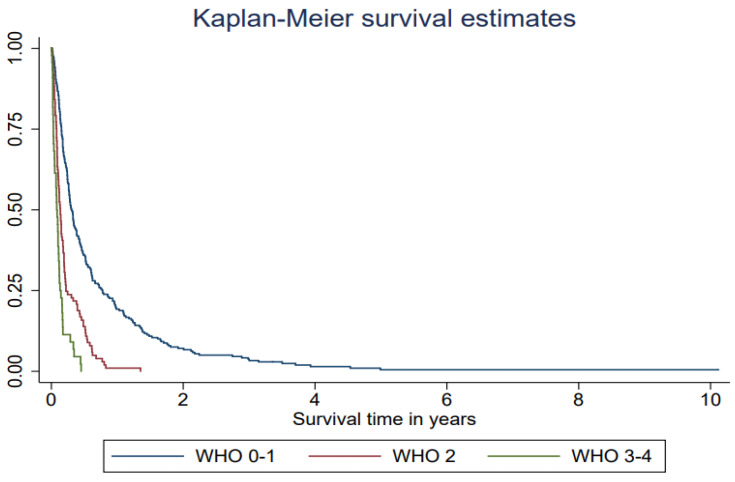
Survival after whole-brain radiotherapy (WBRT) among lung cancer patients (*n* = 384) with brain metastases in Stockholm receiving WBRT at Karolinska University hospital 2008–2019 in relation to performance status (WHO score) one week before WBRT.

The median survival after treatment with WBRT was 2.4 months (interquartile range (IQR) 1.2–6.2 months) (Table 2, Figure 1). The most powerful predictor for survival was performance status. Lung cancer patients who had a PS WHO score of 2 survived a median of 1.6 months from the date of start of WBRT and their HR was over two (HR = 2.33, 95% CI 1.82–3.00). Patients who had a PS WHO score of 3–4 survived a median of 1.0 months, which is the equivalent to a nearly 5-fold increased hazard rate (HR = 4.69, 3.31–6.64) compared with patients with a good performance status, WHO score 0–1 (Table 2, Figure 2). Being admitted to a hospital or a specialized palliative ward before WBRT was associated with higher mortality also in the adjusted models (Table 2). The hazard ratio was significantly higher if the patients had received 2–6 medical oncological treatments before WBRT but not in the model adjusted for performance status. In the ad hoc analysis with the subgroups treated with SRT or surgery in the history before WBRT, the median survival time was 5.5 months in the surgery subgroup; however, this was only 14 patients. 

### 3.4. Health Care Level after Treatment with WBRT

Eighty-four patients (22%) could never be discharged from the hospital after treatment with WBRT (Table 3). Among the group of patients who were admitted to the hospital one week before WBRT, 66 (66%) were not able to come home again, while this was true for 18 patients (7%) in the group of patients who were at home one week before WBRT. In patients with a PS WHO score of 0–1 prior to WBRT, 217 (91%) came home again. In patients with a PS WHO score of 2, 65 (64%) came home again, and in patients with a WHO score of 3–4, 16 (36%) came home again. 

**Figure 3:**
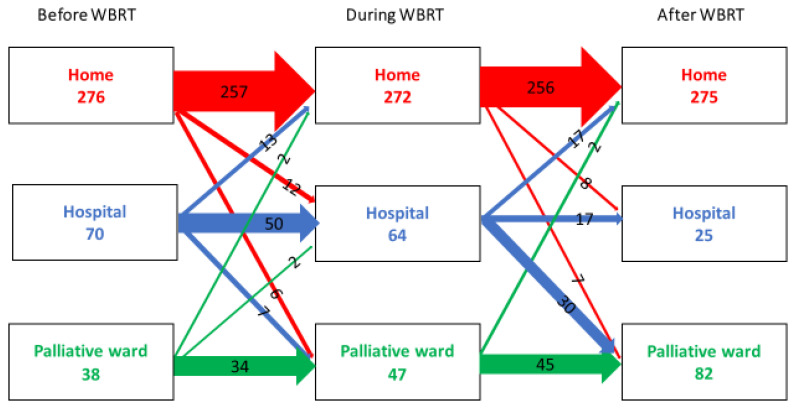
The flow between different health care levels before, during, and after WBRT in a cohort of lung cancer patients (*n* = 384) in Stockholm between 2008–2019.

There were some changes in levels of care in relation to WBRT. Most of the patients at home the week before treatment (*n* = 276) could remain at home during and after the treatment. This was also true for the patients admitted to a specialized palliative ward before treatment (*n* = 38). They stayed in the palliative ward during and after treatment. However, patients who were admitted to the hospital the week before treatment (*n* = 70) were, to a large extent, discharged to a specialized palliative ward or home during or after WBRT. The specialized palliative wards more than doubled the admitted patients the week after WBRT (Figure 3). In the logistic regression model adjusted for the calendar period of WBRT and age, poor performance status was a strong factor associated with not coming home. This was also true for the health care level; patients admitted to the hospital or specialized palliative ward the week before WBRT had higher odds of not coming home again OR 37.15 (95%CI 13.46–102.52) (Table 4). Statistically significant positive predictive factors for coming home again were gender, with greater odds for men even in the adjusted models OR 0.37 (95% CI 0.20–0.68), as well as marital status, where cohabitating patients without children at home had greater odds of coming home, OR 0.29 (95% CI 0.16–0.53). A tendency towards a greater possibility of coming home again was also seen in later periods, 2014–2016 and 2017–2019, even if the OR were not statistically significant. 

## 4. Discussion

In the present single-center population-based cohort study of all lung cancer patients with metastases to the brain treated with WBRT, the overall median survival was short, 2.4 months from the start of treatment with WBRT. One in five patients in this cohort, more women than men, were not able to return home again after treatment with WBRT. The prognosis for lung cancer patients with brain metastases is generally poor. In this study, we did not have the opportunity to compare with patients who did not receive WBRT for brain metastasis, so we cannot draw any conclusions regarding the efficacy of the treatment. 

However, the short survival time is in line with results from a recent study on SRT and WBRT in a Norwegian cohort covering the period from 2006 to 2018, where the median overall survival in the WBRT-group was 3.0 months [36].

We observed, as expected, a strong association between poor PS WHO score and short survival. Lung cancer patients with PS WHO score 3–4 had a median overall survival of only 1.0 months. The odds of not coming home after treatment with WBRT were affected by PS and the health care level one week before treatment with WBRT and by gender. The odds of not coming home again were higher for women than for men and the difference was statistically significant. The odds of not coming home again were also affected by marital status; patients living alone or with children living at home ran a higher risk of prolonged stay in hospital or palliative wards.

Patients with small and few metastases to the brain can be treated with neurosurgery, sometimes with adjuvant radiation therapy afterward or with stereotactic radiotherapy [7]. Stereotactic radiosurgery is recommended for patients who cannot go into neurosurgery, with at most 4–5 brain metastases (Less than 3 cm in diameter) and with no or only a few symptoms. These developments of more local treatments in combination with guidelines advocating best supportive care over WBRT in patients with a survival time of fewer than 3 months changed treatment practice at the Karolinska University hospital already early in the 2010′s, resulting in a decrease in the use of WBRT as single treatment over time during our studied time period, from 175 2011–2013 to 88 2014–2016. In 2016, the QUARTZ study was published [20]. This was a randomized trial addressing the efficacy of best supportive care alone versus WBRT together with best supportive care in patients with NSCLC. The results concluded that WBRT can be omitted and that patients can be treated with the best supportive care alone, without an important reduction in either overall survival or QoL. These results may have further affected the use of WBRT and reduced the WBRT-treated patients observed in our cohort, with only 25 patients treated in 2017–2019 due to a change in treatment practice at the Karolinska University Hospital. This is in line with another study on a similar Stockholm cohort from the same time period that showed a reduction in the use of all radiotherapy in lung and pancreatic cancer patients in Stockholm in the last 30 days of life when comparing 2017 and 2010 [37]. Unfortunately, the selected group of patients in the present study treated with WBRT in the latter time period did not have a significantly better survival compared to earlier time periods. From these real-life data, we can conclude that the selection of patients for WBRT emphasizing PS and introducing scoring systems in the clinic did not result in better survival over time. However, while one-quarter of patients in the earlier time periods (2008–2010, 2011–2013) did not come home after treatment, only 14 and 12% of patients did not come home in 2014–2016 and 2017–2019, respectively. Our data nevertheless suggest that we need to be better at using existing tools to select patients that will benefit from treatment or possibly that the ones currently in use today are difficult to implement in the clinic and that easier tools, therefore, need to be explored. However, for a patient with good performance status and disease under control outside the CNS, WBRT may very well be beneficial. The goals for treatment with WBRT are symptom relief and reduced need for steroids, as well as an increase in life expectancy. Patients with fewer symptoms may have less need for palliative care during a period after treatment with WBRT. Theoretically, the benefits of radiotherapy, in general, may first be experienced after a few days or after up to a few weeks after the treatment with WBRT. In light of this delayed effect of WBRT for symptom control of the disease, our results support that patients with poor performance status and that have a short, expected survival may benefit more from refraining from WBRT than from the treatment, well in line with previous studies and real-life experiences [36,38].

Existing scoring systems to predict the prognosis for patients with brain metastases, such as Radiation Therapy Oncology Group (RTOG) recursive partitioning analysis (RPA) [15], graded prognostic assessment (GPA) [16], and diagnosis-specific GPA (Lung-molGPA score) would have been possible to use in this cohort of lung cancer patients. They have not been used systematically, more in occasional cases. More widespread use of these scoring systems would probably have spared some patients’ treatment with WBRT. 

Health care level after treatment with WBRT is expected to differ by patient characteristics and survival, but also by external factors such as family situation and access to specialized palliative home care. In the Stockholm area, the access to specialized palliative home care is high. Therefore, we found it surprising to note that as many as 20% of all patients in the cohort were not able to come home again from the hospital after treatment with WBRT. For many lung cancer patients who received WBRT in the present study, the best supportive care and a dialogue addressing both the patient’s and the family’s aim for care at the end-of-life situation would have been preferable. Toxic side effects due to treatment with WBRT could be avoided and time spent in hospital for the patients due to treatment would then be saved. Deaths in hospitals are common among cancer patients in western counties, even though their own homes are the most preferred place for dying, as shown in a small Swedish study from 2019 [39]. In this retrospective study of 456 deceased patients, they collected data from the medical records of the patients who were admitted to one of the specialized palliative home care units in the Stockholm region in 2017. Data on several variables were collected from the medical files, such as age, diagnosis, marital status, actual, and preferred place of death. In the cohort, 154 patients (34%) had preferred the place of death in the medical files, 116 (75%) had expressed ending their life in their own homes, and 38 (25%) in a specialized palliative ward. Of the patients who had expressed a preferred place of death, 80% (*n* = 123) had their wish fulfilled, and there were no differences between men and women. In another Swedish review article from 2017, Nilsson et al. conducted a systematic review of 23 papers that studied the patient’s wishes in the end-of-life situation and found a preference for home deaths in 59.9% (39.7–100%) of these studies. The preferred place of death and the actual death place among these studies were significantly different (*p* < 0.05) [26]. In the present study, we found that cohabitating lung cancer patients with no children at living home were discharged from the hospital more often than lung cancer patients in other family situations. These patients may have a strong wish to go home and be discharged from the hospital and may thus have a higher ability to communicate their wishes together with their families. Our findings suggest some overtreatment of WBRT for terminally ill lung cancer patients with brain metastases during the studied time period. The results encourage the use of validated scoring systems, such as the diagnosis-specific GPA (Lung-molGPA score), to help the clinician predict the prognosis and choose the most optimal strategies together with the patients. Many of the patients probably did not gain a better QoL due to the treatment and may have spent the last time in the hospital or traveling to the radiotherapy department for daily treatments. For these palliative patients, dialogues about the patient’s wishes for health care during the terminal period should consequently occur. 

There is, of course, also a health economical aspect of using health care and treatments on patients that will not be beneficial to them. 

The strengths of the present study include the population-based cohort of all lung cancer patients with brain metastases treated with WBRT in the Stockholm area from 2008 to 2019 and the use of prospectively recorded clinical information from medical files. Although the cohort is heterogeneous, including different histological types of lung cancer, with different numbers of brain metastases, we think that the broad inclusion criteria make the results more generalizable. Limitations were the small number of patients from one single center, which reduces precision in some analyses. In some cases, we also used indirect estimation of information, such as the variable PS WHO score based on notes in the medical file. 

## 5. Conclusions

In this clinical cohort study of all lung cancer patients with brain metastases treated with WBRT, one in five patients could not be discharged from the hospital after treatment. There were significantly more women that did not come home again. 

Patients that can stay at home and have a good performance status (WHO score 0–1) seem to benefit more from WBRT than those with poorer PS. Patients that need care in a hospital or specialized-palliative-wards and have PS WHO scores of 3–4 seem to benefit less from treatment with WBRT compared to those with WHO score of 0–1.

## Figures and Tables

**Table 1:** Whole-brain radiotherapy (WBRT) for lung cancer patients with brain metastases in Stockholm, Sweden 2008–2019 (total N = 384).

Variable	N (%)
**Gender**	
Women	211 (54.8)
Men	173 (45.1))
**Marital status**	
Cohabitation, no children at home	204 (53.3)
Cohabitation with children at home	15 (3.9)
Alone with children at home	3 (0.26)
Alone	161 (42.0)
Missing	1
**Stage M (Stage IV)**	
M0	88 (22.9)
M1	291 (75.8)
**Histology**	
SCLC	63 (16.4)
Adenocarcinoma	265 (69.0)
Squamous cell	26 (6.8)
Large cell	11 (2.9)
NOS/Mixed	4 (1.0)
Sarcoma	1 (0.3)
Unclassified	9 (2.3)
Missing	5 (1.3)
**EGFR status**	
Positive	35 (9.1)
Negative	135 (35.2)
Missing	214 (55.7)
**ALK status**	
Positive	12 (3.1)
Negative	78 (20.3)
Missing	294 (76.6)
**KRAS status**	
Positive	18 (4.7)
Negative	35 (9.1)
Missing	331 (86.2)
**Adjuvant treatment (radio, chemo, or both)**	
Yes	56 (14.6)
No	328 (85.4)
**Brain metastases at time of diagnosis**	
Yes	177 (46.1)
No	207 (53.9)
**Palliative medical oncological treatment**	
Yes	264 (68.8)
No	120 (31.3)
**No of palliative medical oncological treatments (min-max)**	0–6
**No of palliative medical oncological treatments**	
0–2	329 (85.7)
3–6	55 (16.7)
**Treatment of brain metastases in the medical history before WBRT**	
Surgery	14 (3.7)
SRT	38 (9.9)
Systemic treatment	9 (2.3)
Surgery + SRT	9 (2.3)
None	311 (81.0)
**Year of WBRT treatment**	
2008–2010	96 (25.0)
2011–2013	175 (45.6)
2014–2016	88 (22.9)
2017–2019	25 (6.5)
**Age at time of WBRT treatment (range 36–91)**	
-49	19 (4.9)
50–69	223 (58.1)
70-	142(40.0)
**Dose of WBRT**	
4 Gy x 5	356 (92.7)
3 Gy x 10	22 (5.7)
Other	6 (1.6)
**Performance status at the start of WBRT (WHO)**	
0	82 (21.4)
1	157 (40.9)
2	101 (26.3)
3	39 (10.2)
4	5 (1.3)
**Finished WBRT treatment**	
Yes	365 (95.0)
No	19 (4.9)
**Health care level one week before WBRT**	
Home	276 (71.9)
Hospital	70 (18.2)
Palliative ward	37 (9.6)
Nursing home	1 (0.3)
**Health care level during WBRT**	
Home	272 (70.8)
Hospital	64 (16.7)
Palliative ward	46 (12.0)
Nursing home	1 (0.3)
**Health care level one week after WBRT**	
Home	275 (70.3)
Hospital	25 (6.5)
Palliative ward	80 (20.8)
Nursing home	2 (0.5)
**Ever home after WBRT treatment**	
Yes	298 (77.6)
No	84 (21.9)

Abbreviations: ALK: Anaplastic lymphoma kinase, EGFR: Epidermal growth factor receptor, Gy: Grey, SCLC: Small cell lung cancer, WBRT: Whole-Brain Radiotherapy, WHO: World Health Organization.

**Table 2:** Survival after treatment with whole-brain radiotherapy (WBRT) among lung cancer patients with brain metastases in Stockholm, Sweden 2008–2019.

Characteristics	Median Survival Months (Range)	Adjusted HR ^1^ (95% CI)	Adjusted HR ^2^ (95% CI)
**Gender** Women Men *At the time of treatment with WBRT* **Age (years)** -49 50–69 70+	2.2 (0.1–60.8) 2.6 (0.1–55.2)	1.0 (ref) 0.94 (0.77–1.16)	1.0 (ref) 0.85 (0.69–1.05)
		
	3.6 (0.7–47.9) 2.8 (0.1–123.1) 2.0 (0.1–55.2)	1.0 (ref) 1.09 (0.68–1.75) 1.59 (0.98–2.57)	1.0 (ref) 1.00 (0.63–1.63) 1.33 (0.82–2.18)
**Marital status** Alone Alone with children at home Cohabitation with no childern at home Cohabitation with children at home **Calendar year** 2008–2010 2011–2013 2014–2016 2017–2019	2.1 (0.1–123.1) 1.6 (0.7–47.9) 2.8 (0.1–60.8) 2.9 (0.1–22.0)	1.0 (ref) 0.47 (0.13–1.68) 0.82 (0.66–1.01) 0.98 (0.53–1.79)	1.0 (ref) 0.39 (0.11–1.39) 0.83 (0.66–1.04) 0.90 (0.48–1.67)
	2.2 (0.1–123.1) 2.5 (0.1–60.8) 2.8 (0.1–47.9) 2.6 (0.3–40.8)	1.0 (ref) 0.85 (0.66–1.09) 1.00 (0.75–1.35) 0.75 (0.48–1.18)	1.0 (ref) 0.86 (0.67–1.11) 1.14 (0.85–1.53) 0.90 (0.57–1.41)
**WBRT dose** 4 Gy x 5 3 Gy x 10 **WHO performance status score** 0–1 2 3–4 **Health care level one week before WBRT** Home Hospital Palliative ward *At primary lung cancer diagnosis*	2.4 (0.1–123.1) 3.5 (0.8–36.4)	1.0 (ref) 0.84 (0.55–1.29)	1.0 (ref) 0.89 (0.58–1.37)
	3.7 (0,1–123.1) 1.6 (0.1–16.5) 1.0 (0.1–5.5)	1.0 (ref) **2.33 (1.82–3.00)** **4.69 (3.31–6.64)**	-- -- --
	3.45 (0.1–60.8) 1.38 (0.1–123.1) 0.85 (0.1–7.5)	1.0 (ref) **2.25 (1.70–2.98)** **3.66 (2.54–5.27)**	1.0 (ref) **1.56 (1.15–2.12)** **2.26 (1.53–3.34)**
		
**Stage** M0 M1 **EGFR** Positive Negative	2.0 (0.1–55.2) 2.6 (0.1–123.1)	1.0 (ref) 1.07 (0.84–1.37)	1.0 (ref) 1.14 (0.89–1.46)
	3.4 (0.1–60.8) 3.2 (0.4–55.2)	1.0 (ref) 1.05 (0.70–1.56)	1.0 (ref) 1.12 (0.75–1.69)
*Palliative treatments before WBRT*			
**Palliative medical oncological treatment** No Yes	1.8 (0.1–40.8) 2.9 (0.1–123.1)	1.0 (ref) **0.74 (0.58–0.93)**	1.0 (ref) 0.92 (0.72–1.16)
**No of palliative chemotherapy regimens** 0–2 2–6	2.3 (0.1–123.1) 3.4 (0.3–47.9)	1.0 (ref) **0.71 (0.52–0.96)**	1.0 (ref) 0.84 (0.61–1.14)

^1^ adjusted for calendar period of WBRT (in 3-year intervals) and age at WBRT (in intervals); ^2^ adjusted for WHO performance status score, calendar period of WBRT (in 3-year intervals), and age at WBRT (in intervals); Results that are statistically significant are marked in bold. Abbreviations: CI: Confidence Interval, EGFR: Epidermal growth factor receptor, Gy: Grey, HR: Hazard ratio, WBRT: Whole-Brain Radiotherapy, WHO: World Health Organization.

**Table 3:** Number and proportion of lung cancer patients with brain metastases coming home or not after whole-brain radiotherapy (WBRT) in relation to performance status score and health care level prior to WBRT.

	Patients That Ever Came Home after WBRT		
	Yes	No	Total
**Total**	298 (77.6%)	84 (21.9%)	384 (100%)
**Health care level one week before WBRT**			
Home	257 (93.1%)	18 (6.5%)	276 (100%)
Hospital/Specialized palliative ward	41 (38.0%)	66 (61.1%)	108 (100%)
**Performance status WHO score before WBRT**			
0–1	217 (90.8%)	22 (9.2%)	239 (100%)
2	65 (64.4%)	34 (33.6%)	101 (100%)
3–4	16 (36.4%)	28 (63.6%)	44 (100%)

**Table 4:** The relative risks (odds ratio, OR) for lung cancer patients with brain metastases of not coming home following whole-brain radiotherapy (WBRT) in relation to clinical and biological characteristics.

Clinical and Biological Characteristics	N (%) of Lung Cancer Patients Not Coming Home	Adjusted OR ^1^ (95% CI)	Adjusted OR ^2^ (95% CI)
**Gender** Women Men	57 (27.0) 27 (15.8)	1.0 (ref) **0.51 (0.30–0.85)**	1.0 (ref) **0.37 (0.20–0.68)**
*At the time of treatment with WBRT*			
**Age (years)** -49 50–69 70+	4 (21.1) 45 (20.3) 35 (24.8)	1.0 (ref) 0.92 (0.29–2.94) 1.30 (0.39–4.16)	1.0 (ref) 0.92 (0.23–3.63) 1.04 (0.26–4.21)
**Marital status** Alone Alone with children at home Cohabitation with no childern at home Cohabitation with children at home	54 (33.5) 1 (33.3) 27 (13.3) 2 (13.3)	1.0 (ref) 1.25 (0.09–17.59) **0.32 (0.19–1.54)** 0.31 (0.05–1.73)	1.0 (ref) 0.70 (0.04–13.29) **0.29 (0.16–0.53)** 0.15 (0.02–1.21)
**Calendar year** 2008–2010 2011–2013 2014–2016 2017–2019	23 (24.5) 46 (26.3) 12 (13.6) 3 (12.0)	1.0 (ref) 1.10 (0.62–1.96) 0.47 (0.21–1.01) 0.42 (0.12–1.55)	1.0 (ref) 1.07 0.43 (0.18–1.01) 0.52 (0.13–2.10)
**WBRT dose** 4 Gy x 5 3 Gy x 10	79 (22.0) 5 (21.7)	1.0 (ref) 1.01 (0.36–2.88)	1.0 (ref) 1.61 (0.53–4.89)
**Performance status WHO score** 0–1 2 3–4	22 (9.2) 34 (34.3) 28 (63.6)	1.0 (ref) **5.10 (2.75–9.45)** **17.48 (8.08–37.84)**	-- -- --
**Health care level one week before WBRT** Home Hospital Palliative ward	18 (6.6) 37 (52.9) 29 (78.4)	1.0 (ref) **16.06 (8.08–31.93)** **60.32 (22.70–160.31)**	1.0 (ref) **10.56 (5.10–21.85)** **37.15 (13.46–102.52)**
*At primary lung cancer diagnosis*			
**Stage** M0 M1	20 (23.0) 64 (22.1)	1.0 (ref) 1.00 (0.56–1.80)	1.0 (ref) 1.14 (0.59–2.19)
**EGFR** Negative Positive	23 (17.0) 7 (20.0)	1.0 (ref) 1.12 (0.41–3.02)	1.0 (ref) 0.95 (0.28–3.24)
*Palliative treatments before WBRT*			
**Palliative medical oncological treatment** Yes No	45 (17.1) 39 (32.8)	1.0 (ref) **0.44 (0.26–0.74)**	1.0 (ref) 0.61 (0.34–1.09)
**Number of palliative oncological treatments** 0–2 2–6	75 (22.9) 9 (16.7)	1.0 (ref) 0.72 (0.33–1.57)	1.0 (ref) 1.04 (0.44–2.49)

^1^ adjusted for calendar period of WBRT (in 3-year intervals) and age at WBRT (in intervals); ^2^ adjusted for WHO performance status score and calendar period of WBRT (in 3-year intervals) and age at WBRT (in intervals). Statistically significant results are marked in bold. Abbreviations: CI: Confidence Interval, EGFR: Epidermal growth factor receptor, OR: Odds ratio, WBRT: Whole-Brain Radiotherapy, WHO: World Health Organization.

## Data Availability

The raw data is available from the corresponding author upon request.
